# Large variety in a panel of human colon cancer organoids in response to EZH2 inhibition

**DOI:** 10.18632/oncotarget.12002

**Published:** 2016-09-13

**Authors:** Martijn AJ Koppens, Gergana Bounova, Paulien Cornelissen-Steijger, Nienke de Vries, Owen J Sansom, Lodewyk FA Wessels, Maarten van Lohuizen

**Affiliations:** ^1^ Division of Molecular Genetics, The Netherlands Cancer Institute, Amsterdam, The Netherlands; ^2^ Division of Molecular Carcinogenesis, The Netherlands Cancer Institute, Amsterdam, The Netherlands; ^3^ Cancer Research UK Beatson Institute, Glasgow, United Kingdom; ^4^ Department of EEMCS, Delft University of Technology, Delft, The Netherlands; ^5^ Cancer Genomics Centre Netherlands (CGC.nl), The Netherlands

**Keywords:** GSK126, colorectal cancer, organoids, polycomb, Tp53

## Abstract

EZH2 inhibitors have gained great interest for their use as anti-cancer therapeutics. However, most research has focused on EZH2 mutant cancers and recently adverse effects of EZH2 inactivation have come to light. To determine whether colorectal cancer cells respond to EZH2 inhibition and to explore which factors influence the degree of response, we treated a panel of 20 organoid lines derived from human colon tumors with different concentrations of the EZH2 inhibitor GSK126. The resulting responses were associated with mutation status, gene expression and responses to other drugs. We found that the response to GSK126 treatment greatly varied between organoid lines. Response associated with the mutation status of *ATRX* and *PAX2*, and correlated with *BIK* expression. It also correlated well with response to Nutlin-3a which inhibits MDM2-p53 interaction thereby activating p53 signaling. Sensitivity to EZH2 ablation depended on the presence of wild type p53, as tumor organoids became resistant when p53 was mutated or knocked down. Our exploratory study provides insight into which genetic factors predict sensitivity to EZH2 inhibition. In addition, we show that the response to EZH2 inhibition requires wild type p53. We conclude that a subset of colorectal cancer patients may benefit from EZH2-targeting therapies.

## INTRODUCTION

Elevated levels of the Polycomb Group (PcG) protein EZH2 are found in a wide range of cancer types, and are often correlated with poor prognosis [1, 2]. Highly differentiated adult tissues generally express *Ezh2* at low levels, while embryonic tissues and highly proliferating tissues have high *Ezh2* expression [3–5]. Reducing cellular EZH2 activity has previously been shown to negatively affect cell proliferation of certain tumor types [6–11]. The advent of high-specificity small molecule inhibitors against EZH2 has reinvigorated the assessment of EZH2 as a potential anti-cancer therapeutic target. Lymphomas with an activating mutation in the catalytic SET domain of EZH2 are strongly affected by treatment with the EZH2 inhibitor GSK126 [9] and clinical trials with EZH2 inhibitors are currently ongoing. However, reducing EZH2 levels has also been shown to have its dangers, as particular myelodysplastic syndromes naturally inactivate *EZH2*, suggesting a tumor suppressor role for EZH2 in this context [12–14]. More importantly, prolonged EZH2 depletion causes glioblastoma cells to acquire a more aggressive phenotype [15]. In addition, KRAS-mutant lung tumors seem to benefit from disruption of the Polycomb Repressive Complex 2 (PRC2), of which EZH2 is a subunit [6, 16]. It is therefore crucial to thoroughly investigate the mutational landscape and transcriptional profile that define sensitivity to EZH2 inhibition. Such studies require a comprehensive overview of the mutations and gene expression patterns that define tumor types and their subtypes. Colorectal cancer (CRC) is one such cancer that has been subject to intense molecular characterization, and has recently been reclassified into four consensus molecular subtypes (CMS) [17]. The recent advances in the intestinal organoid culture system have made it possible to *in vitro* propagate human CRC tumors without losing the genetic and expressional identity of the original tumor, while the diversity that is found in CRC is largely maintained [18, 19]. These advantages over conventional cell lines and mouse models, makes the organoid culture method an excellent tool to assess the drug response patterns across the different CRC subtypes.

So far, a limited number of cancer types have been demonstrated to respond well to treatment with EZH2 inhibitors. Particularly sensitive tumors are those with mutated SWI/SNF [8] or containing an activating mutation in the SET domain of EZH2 [9]. As screening methods to discover cancers sensitive to EZH2 inhibition are principally done using conventional cancer cell lines, it is possible that this two-dimensional (2D) cell culture system does not properly represent the physiology of the tumor, which could impair discovery of cancers targetable with EZH2 inhibitors. Another possible cause for the lack of response by conventional cell lines could be the use of high-passage cell lines in such screens.

In this exploratory study, we investigated the response of a panel of twenty well-characterized human CRC organoid lines derived from colon cancers [18] to treatment with the EZH2 inhibitor GSK126 over a course of multiple weeks. The setup of these GSK126-response assays (termed “viability assays” in this manuscript) was different from high-throughput drug screens in three ways. First, testing a single drug allowed us to treat larger numbers of organoids per dose, thus reducing noise in quantifying organoid viability. Second, we determined treatment time for each organoid line by the growth rate rather than having the same treatment time for all organoids, which allowed slowly growing organoid lines to develop a proper response. Third, by treating all organoids for at least nine days, and treating a subset of eight organoid lines for a prolonged period of time, long-term effects beyond immediate response could be assessed.

We demonstrate that this panel displays a wide range of sensitivity to EZH2 enzymatic inactivation. By performing a comprehensive analysis, we explored associations of GSK126 response with mutation, gene expression and drug response data that have previously been measured in these organoids [18]. We found that response correlates with the mutation status of a number of genes, including *ATRX* and *PAX2*, with expression of the pro-apoptotic gene *BIK* as well as with sensitivity to the MDM2 inhibitor Nutlin-3a. This study is the first to investigate the response of a panel of human CRC organoids to treatment with the epigenetic drug GSK126, the results of which demonstrate various degrees of response within the group of organoids, thereby providing a rationale for further investigation into its use as a therapy to treat CRC. In addition, we reveal a set of features that may predict patient response to EZH2 inhibition.

## RESULTS

### *EZH2* expression is increased in CRC organoids

**Figure 1:**
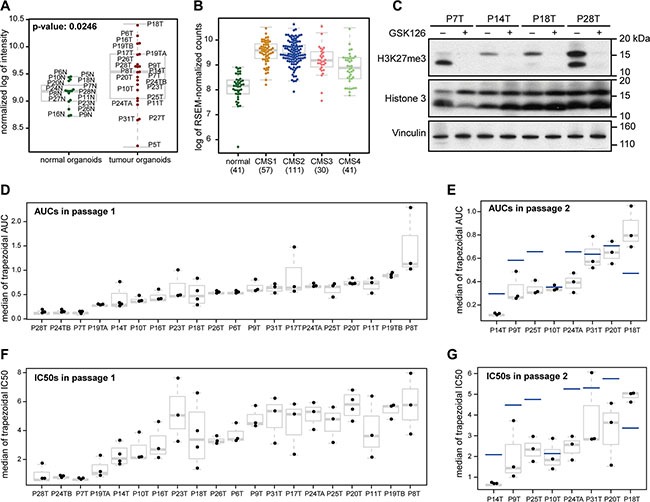
Organoids from different CRC tumors respond differently to chemical EZH2 inhibition (**A**) *EZH2* expression in normal colonic organoids and CRC organoids. Both the mean and the variance of *EZH2* expression are higher in tumor organoids as compared to normal organoids. (**B**) *EZH2* expression in normal colon and the different molecular CRC subtypes. Expression data were taken from CRC RNA-sequencing samples from the TCGA database. CMS4 tumors express *EZH2* significantly lower than other subtypes: comparing CMS1 and CMS4, *p*-value: 1.2 * 10^−7^, comparing CMS2 and CMS4, *p*-value: 1.6 * 10^−7^, and comparing CMS3 and CMS4, *p*-value: 0.0277. *EZH2* expression in normal colon tissue was lower than tumor tissue (compared with CMS1-4 combined, *p*-value: 2.0 * 10^−15^). The numbers below the x-axis indicate the number of samples. (**C**) Western blot of four organoid lines that were treated with 4 μM GSK126 and harvested after fourteen days. H3K27me3 is strongly reduced in GSK126-treated versus DMSO-treated samples. (**D**, **E**) Box plot showing the trapezoidal AUCs for all replicates of 20 CRC organoids in passage 1 (D) and eight CRC organoids in passage 2 (E). **(F**, **G**) Box plot showing the trapezoidal IC50s for all replicates of 20 CRC organoids in passage 1 (F) and eight CRC organoids in passage 2 (G). Horizontal gray lines within boxes demarcate the medians, boxes delineate the middle 50% of the data, and whiskers mark 25% and 75% quartiles. The horizontal blue lines in (E and G) demarcate the medians from passage 1 for the same organoid line.

*EZH2* expression levels are often elevated in colorectal tumors [20]. In order to determine whether *in vitro* cultured organoids displayed a similar pattern, we evaluated *EZH2* expression levels in the panel of 22 CRC organoids and their normal tissue counterparts [18]. Normal colon-derived organoid lines had a narrow range of *EZH2* expression as compared to CRC organoid lines, of which most had higher *EZH2* expression levels than any of the normal colon-derived organoids (Figure 1A). Three CRC organoid lines had particularly low *EZH2* levels, two of which have originally been classified into the stem-like molecular subtype (which corresponds to CMS4 in the CMS classification [17]) and were unable to be propagated during the initial expansion of the panel. Accordingly, *EZH2* expression in CMS4 samples from a TCGA cohort of 239 CRC samples was lower than in other CMS subtypes (Figure 1B). As CMS4 tumors are typified by a high stromal content, this lower expression may also be due to relative *EZH2* transcript dilution by low *EZH2*-expressing stromal cells in CMS4 RNA samples. Unfortunately, as organoid cultures from CMS4 tumors have a low success rate, we were unable to validate whether low *EZH2* expression is a common feature of CMS4 organoids. Further, normal colon tissue expressed *EZH2* at lower levels than CRC tissue, which is in line with the expression data from organoids. Taken together, these results show that the majority of CRC organoids have increased *EZH2* expression, which is in concordance with previously published data on CRC tissue.

### Large variability in growth response between CRC organoid lines after exposure to the EZH2 inhibitor GSK126

In order to determine whether EZH2 is required for growth of CRC or one of its subtypes, we treated the 20 colon cancer organoid lines with the EZH2 inhibitor GSK126. First, reduction of Histone 3 Lysine 27 trimethylation (H3K27me3) was validated after 14 days of GSK126 treatment for four randomly chosen organoid lines (Figure 1C). Next, we performed viability assays for all 20 organoid lines. 2000 single cells were seeded per well and grown for three days to form small organoids. They were then subjected to different GSK126 concentrations between 1 μM and 8 μM, as previous studies have shown the drug's optimal EZH2 inhibition activity to be within this range. After a certain number of days (this varied between organoid lines), one of the conditions had reached growth-limiting size, which was typically the untreated control. At this stage, called passage 1, the organoids were trypsinized and single cells of each condition were counted. For eight organoid lines, we reseeded 2000 cells of each concentration and maintained the same treatment regimen until again organoids had reached growth-limiting size (between 21 and 30 days), after which the organoids were trypsinized and single cells were counted (passage 2). Representative images of organoid lines at passage 1 and passage 2 are depicted in Supplementary Figure 1A and 1B, respectively.

Viability was computed for passage 1 and passage 2 following two different approaches, as explained in the Methods, using the trapezoid rule, as well as curve fitting (Supplementary Figure 2A and 2B). For each approach IC50 values and area under the curve (AUC) values were computed. The four resulting viability measures showed good correlation (Supplementary Figure 2C). We observed a large variability in response to GSK126 in this CRC organoid panel for passage 1 (Figure 1D, 1F). A few organoid lines were very sensitive to EZH2 inhibition, while others were relatively resistant. The degree of response was maintained for three organoid lines in passage 2, while four became more sensitive and only P18T became more resistant (Figure 1E, 1G).

Interestingly, we noticed that some organoid lines grew faster when treated with low concentrations of GSK126, i.e. exhibiting a non-monotonous response (Supplementary Figure 2A and 2B). We grouped the lines into monotonous – whose viability only decreases with increasing GSK126 concentrations – and non-monotonous – with a positive growth response at low concentrations that is at least 1.5 times of that of untreated control – responding organoids for both passages and compared molecular features for the two groups, as explained in the Methods. There were no strong differences between the two groups in the mutation data. Gene expression comparison between monotonous and non-monotonous responding organoid lines yielded four genes that were differentially expressed between the two groups. *KLK6* was significantly less expressed in non-monotonous responders, while *DMRT2*, *ALDH1L2* and *RAB39A* expression levels were higher in this group (Supplementary Table 1A).

### Adherent growth of two sensitive CRC organoid lines does not render them resistant

**Figure 2:**
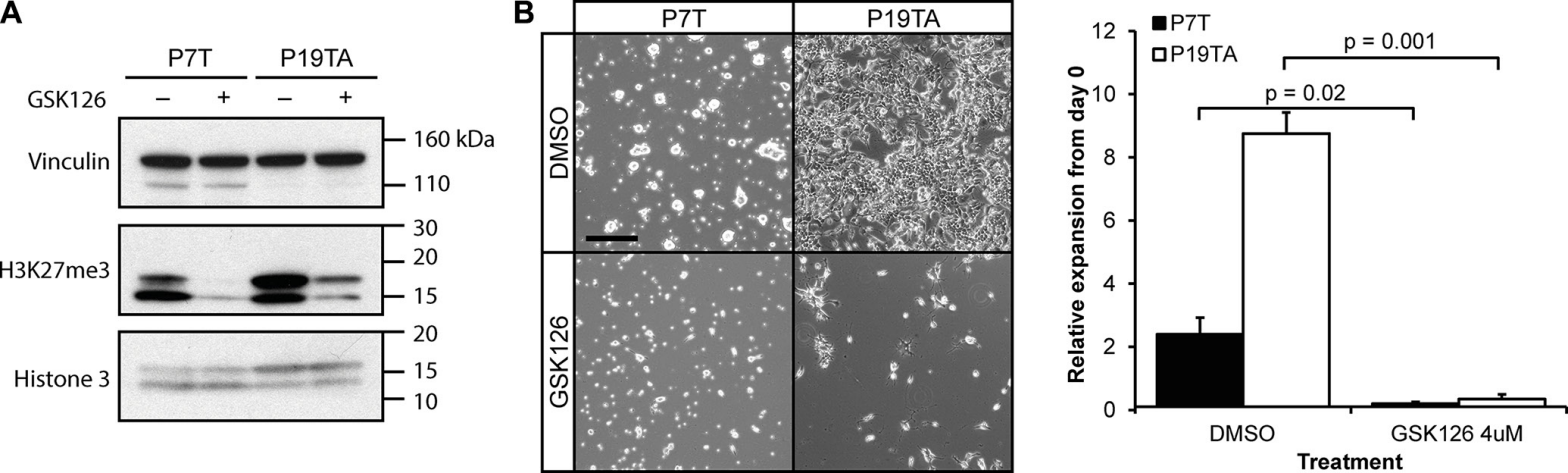
Adherently cultured GSK126-responsive organoids remain sensitive to EZH2 inhibition (**A**) Western blot confirming reduction of H3K27me3 in adherent organoid cultures upon 14 days of GSK126 treatment. (**B**) Left panel: microscopy images of adherently cultured P7T and P19TA, treated with either 4 μM of GSK126 or DMSO for fourteen days. Right panel: bar plot showing the average fold expansion in number of cells after fourteen days of DMSO or GSK126 treatment. Error bars indicate standard deviation. Scale bar: 100 μm, same magnification applies to all images in (B).

To investigate whether organoids would respond differently to EZH2 inhibition when cultured in adherent conditions, we transitioned two of the most sensitive organoid lines, P7T and P19TA, to an adherent state by culturing them on laminin-coated plates. These organoid-derived adherent cell lines were then treated for 14 days with 4 μM of GSK126, a concentration at which both corresponding organoid lines were growth inhibited. Robust reduction of H3K27me3 was confirmed by western blot (Figure 2A). Strikingly, both cell lines were strongly inhibited by EZH2 inhibition and no resistant colonies grew out during the two weeks of treatment (Figure 2B). These results suggest that transitioning GSK126-responsive organoids to an adherent state does not render them resistant to EZH2 inhibition. Of note, we were unable to generate adherently growing cell lines from organoid lines that are relatively resistant to EZH2 inhibition.

### Setup of association analysis between response to EZH2 inhibition and molecular features

To gain insight into the properties that might cause sensitivity or resistance to EZH2 inhibition, we associated measured molecular features of the tested organoid panel [18] with GSK126 response. In order to increase robustness of the identified associations, the same analyses were performed with all four computed measures of GSK126 response. The same analyses were performed for passage 1 and for passage 2. As explained in the methods section, we performed univariate associations (single features with multiple test correction) and multivariate associations (combinations of features).

In particular, we tested for associations between GSK126 response and CRC subtype status, hyper-mutated status, mutation status and gene expression data. Due to the small sample size (20), many weak as well as cohort-specific effects were observed. This is especially true for passage 2, which contains only 8 samples. We therefore focus only on the strongest observations that also recur in different parts of the analysis.

### Association with subtypes and hyper-mutant status

No significant effects were observed in the CRC subtype associations using either the previously published subtyping by Sadanandam et al. [21] or the CMS subtyping [17] (Supplementary Table 1B). Four of the six hyper-mutants are among the most sensitive organoids to the EZH2 inhibitor (P7T, P24TB, P19TA and P10T), but the remaining two (P24TA and P19TB) are among the resistant samples, resulting in a weak effect with none of the viability measures significantly associating with hypermutated status (Supplementary Figure 3A).

### Response to EZH2 inhibition associates with *ATRX* and *PAX2* mutation status

We next tested for association between GSK126 response and mutation status. Before analyzing all mutations reported in the panel, we looked at *EZH2* and other PcG gene mutations. There is only one *EZH2*-mutant in the organoid panel: P10T (p.N361T missense SNP), which is a hyper-mutated line and among the sensitive organoids in both passages. This rare mutation is outside of the catalytic SET domain or other well-defined domains. Mutations that could affect other Polycomb Group genes were also present in one of the sensitive organoid lines: *BMI1* bears a p.F280L missense mutation in P10T and P7T has an intergenic deletion 8367bp upstream of *EZH1*. Although all PcG mutations occur in sensitive organoid lines, each occurrence is in one sample only and cannot be analyzed statistically.

**Figure 3:**
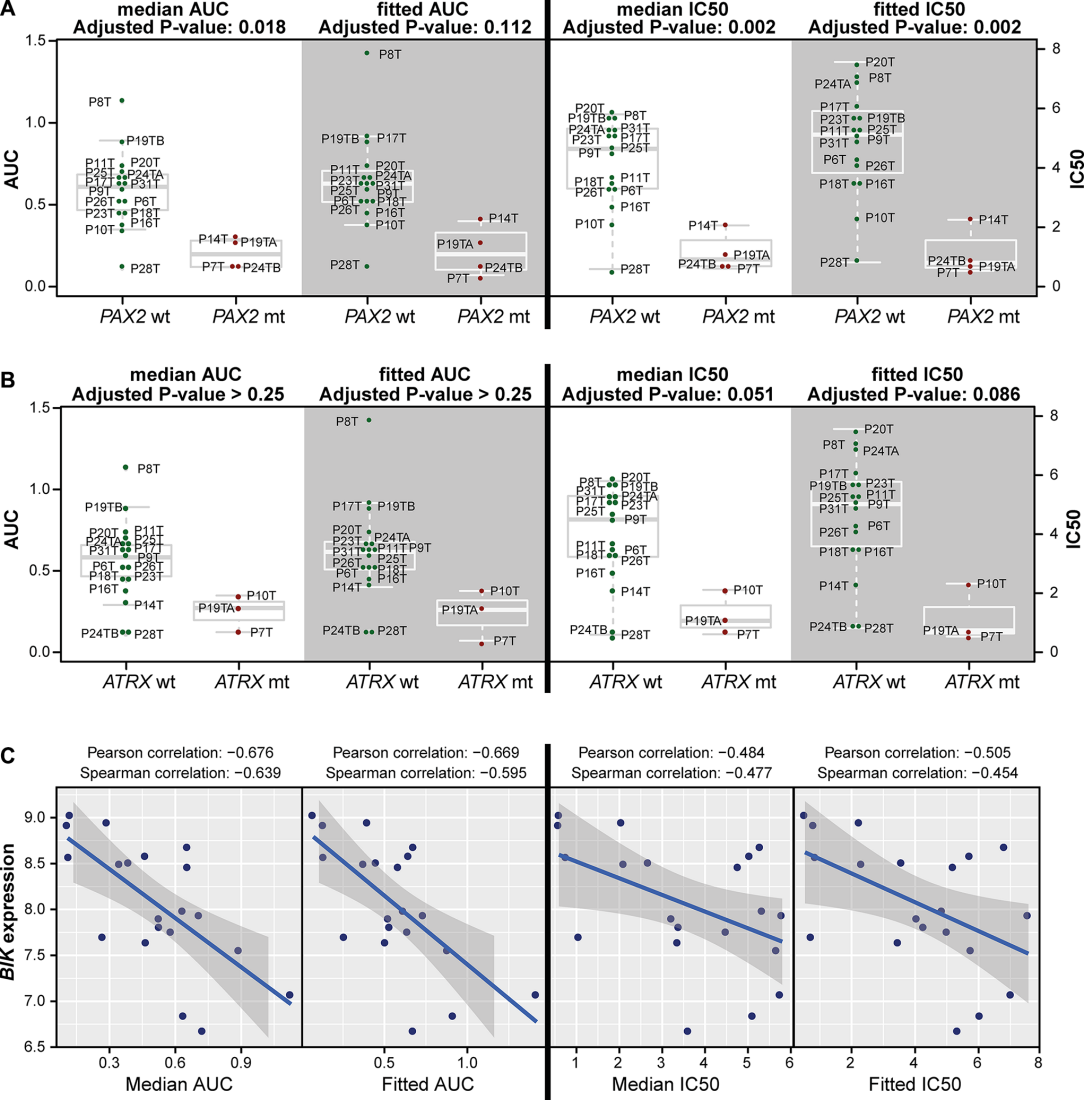
Response to EZH2 inhibition associates with mutation status and BIK gene expression **(A)** PAX2-mutant organoid lines are significantly more sensitive to GSK126 according to all four viability measures in passage 1. **(B)** ATRX-mutant organoid lines are significantly more sensitive to GSK126 for two out of four viability measures for passage 1. In A and B, four panels of box plots are shown (AUC: left two panels, IC50: right two panels; median: white background, fitted: gray background) comparing organoid lines that are wild type (left group, green dots) with organoids that are mutant (right group, red dots) for the indicated genes. Horizontal gray lines within boxes demarcate the medians, boxes delineate the middle 50% of the data, and whiskers mark 25% and 75% quartiles. **(C)** BIK expression inversely correlates with GSK126 response for three out of four viability measures (fitted IC50, and median and fitted AUC) in passage 1.

For the analysis considering all mutations reported in the panel, we grouped collinear mutations, i.e. mutations present in exactly the same set of organoid lines. Also, a mutation was analyzed only if it is present in at least two samples. Finally, mutations that occur in hyper-mutant samples only were flagged, as they are more likely to be passenger mutations. Large collinear groups were also flagged, as there is no way to pinpoint a driver mutation without prior knowledge. Lastly, mutations had to significantly associate with response for at least two viability measures out of four. Using all mutations in the data set, we found for passage 1 the mutation status of a number of genes to be associating with response to EZH2 inhibition (Supplementary Table 1C). *PAX2* is among these genes, where mutant samples are more sensitive (Figure 3A). We noticed that several associating features had a high number of collinear mutations and many involved only hypermutated organoid lines. To specifically obtain the meaningful mutations, we reran the analysis using a selected list of genes that are often mutated in CRC [22] (Supplementary Table 1D) and found that mutant *ATRX* associated with sensitivity (Figure 3B). In fact, ATRX was also among the results of the first analysis that includes all mutations in the cohort, but it was collinear with seven other genes (Supplementary Table 1C). For passage 2, no association was found between response and mutation status of any gene. Multivariate analysis yielded only weak associations between mutation combinations and response (data not shown).

### GSK126 response inversely correlates with BIK expression

Next, we analyzed correlations between response to EZH2 inhibition and gene expression, for which the top 2430 highly-varying genes (top 11.2%) were used. Notably, response does not correlate with *EZH2* expression. Again, genes whose expression significantly correlated with GSK126 response for multiple viability measures and analyses are reported here (Supplementary Table 1E). The best correlation for multiple viability measures was for *BIK* (Figure 3C) which encodes for a protein involved in apoptosis [23]. The correlation is inverse, i.e. organoid lines with high *BIK* levels are sensitive to GSK126 treatment. The multivariate gene expression associations did not yield any significant results. Although analysis of passage 2 yielded many genes whose expression highly correlated with response, we decided not to report these due to the low number (8) of tested organoid lines.

### Sensitivity to EZH2 inhibition correlates with activity of the p53 pathway

**Figure 4:**
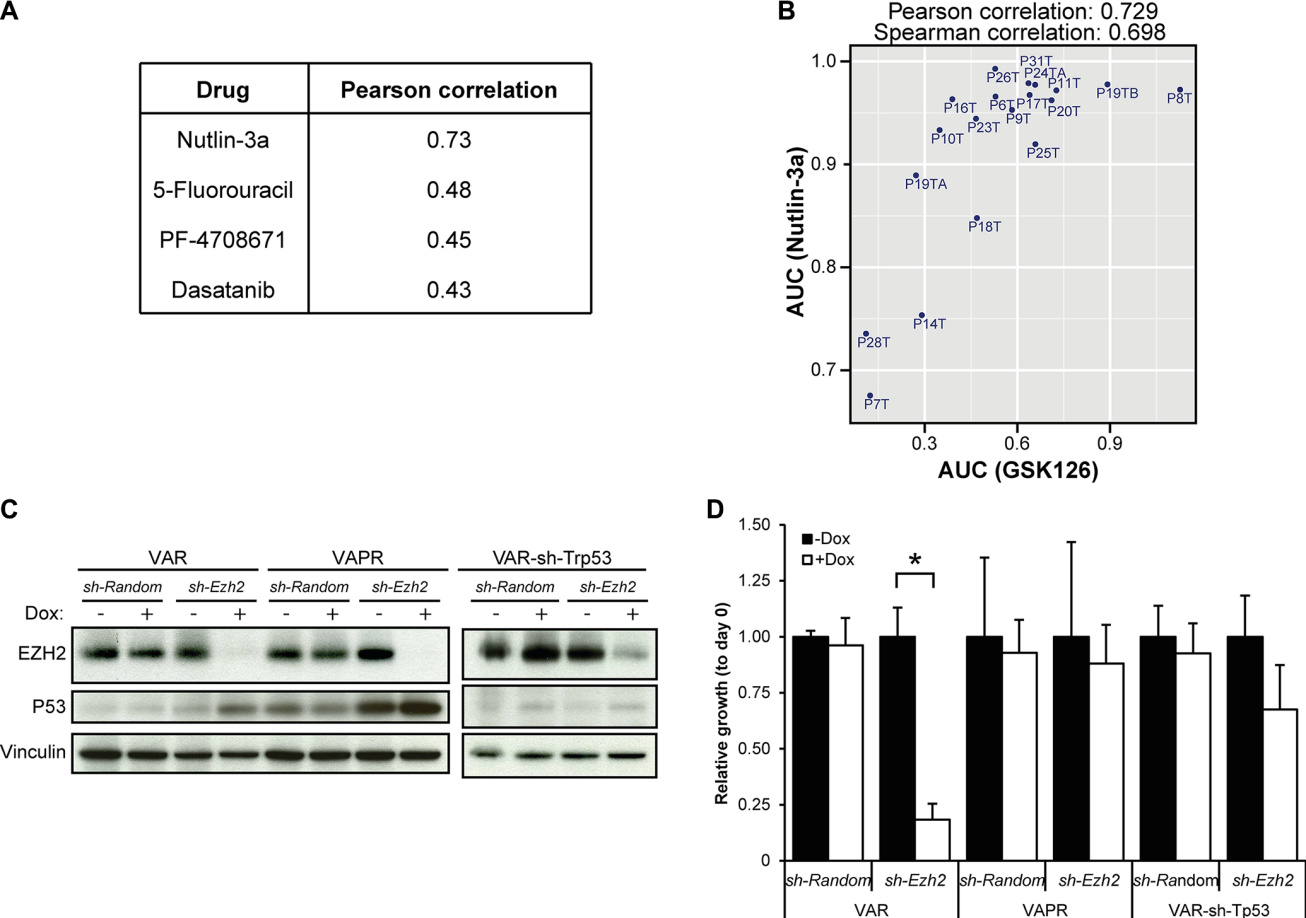
Sensitivity to EZH2 inhibition is correlated with activity of WT-p53 signaling **(A)** The four highest correlating drug responses with response to GSK126 (heatmap of Pearson correlations between all pairs of 84 drugs is presented in Supplementary Figure 4A). **(B)** Scatter plot showing the high correlation between the AUCs of Nutlin-3a and GSK126. **(C)** Western blot showing efficient Ezh2 KD and increased levels of Trp53 in Ezh2 KD organoids. **(D)** Average relative growth of VAR, VAPR and VAR-sh-Trp53 tumor organoids with or without Ezh2 KD, normalized to not-induced samples (”-Dox”, black bars). Error bars indicate standard deviation. (*n* = three independent experiments; 1-way ANOVA followed by Post hoc tests; *P = 6.7*10^−4^, only *P*-values < 0.05/12 are depicted).

For 19 of the 22 CRC organoids, their responses to 83 drugs have been previously measured [18]. Besides genetic and transcriptional features, these drug responses can also provide insight into the mechanisms of response to EZH2 inhibition. We compared response to GSK126 with the other drug responses (Supplementary Figure 4A). Sensitivity to EZH2 inhibition highly correlated with sensitivity to Nutlin-3a (Figure 4A, 4B), and to a lesser extent with sensitivity to 5-FU, PF-4708671 and Dasatinib (Figure 4A). The compound Nutlin-3a prevents binding of MDM2 to p53, which results in p53 stabilization and subsequent activation of downstream effector pathways [24]. Consequently, Nutlin-3a is most effective in cells with an intact p53 pathway and, accordingly, in the original proof-of-concept drug screen mutation of *TP53* was associated with resistance to Nutlin-3a. Yet, in our own analysis mutation of *TP53* was not significantly associated with resistance to GSK126. However, the p53 pathway can be disrupted by other means than *TP53* mutation [25, 26]. The degree of response to Nutlin-3a, rather than *TP53* mutation, may therefore be seen as a measure for how intact the p53 pathway still is. To test whether sensitivity to EZH2 inhibition depends on an unperturbed p53 pathway, we knocked down *Ezh2* in murine tumor organoids with defined genotypes. These organoid lines were originally derived from the small intestines of *VillinCre*; *Apc^f/f^*; *KRas^G12D^* (VAR) and *VillinCre*; *Apc^f/f^*; *KRas^G12D^*; *p53^−/R172H^* (VAPR) mice. Organoids were subsequently transduced with Doxycycline-inducible constructs encoding either a short hairpin against *Ezh2* or a control short hairpin (*sh- Random*) (Figure 4C). Upon treatment with Doxycycline, *Ezh2* knockdown (KD) VAR organoids were growth arrested, while *Ezh2*-KD VAPR organoids showed no effect on growth (Figure 4D). The growth arrest in VAR was rescued by additional knockdown of *Trp53* (Figure 4D and Supplementary Figure 4B). Interestingly, p53 protein levels increased upon *Ezh2* KD in VAR as well as in the VAPR organoids that only have mutant p53 (Figure 4C). This suggests that the detrimental effects of EZH2 inhibition are mediated through stabilization of p53 protein.

## DISCUSSION

The panel of human CRC organoids tested in our study shows a large variability in response to treatment with the EZH2 inhibitor GSK126, providing a rationale for using high-specificity EZH2 inhibitors as anti-cancer therapy for a subset of CRC patients. In the past years, several other highly specific EZH2 inhibitors have been characterized [8, 9, 27, 28], three of which were shown to cause highly similar changes in the gene expression profile when used on a lymphoma cell line [27], indicating a common mechanism of action both on-target and off-target. This is not entirely surprising, as their molecular structures are very similar.

With the organoid culture system becoming an established technique, its application in various lines of research, among which are small and large scale drug testing [18, 29–32], is being further developed. The setup of our viability assay made it possible to monitor the effects of prolonged EZH2 inhibition and allowed slowly growing organoids to develop a response. We did not use a fixed treatment time, but rather timed it by the speed of organoid growth. Consequently, response to EZH2 inhibition could be better compared between organoid lines with different growth rates. However, any cytotoxic effects of GSK126 treatment impair this comparison as it aggravates the response of slowly growing organoid lines with a longer treatment time. Nevertheless, although demethylases do contribute to removal of H3K27me3 from the chromatin, cellular decline in H3K27me3 upon PRC2 ablation has been shown to be linked to cell division [27, 33].

Using four methods to compute the degree of response to GSK126, we identified different dynamics in the organoids’ responses to EZH2 inhibition. In general, the different viability measures highly correlated with one another, except for two outliers. While P8T responds well to the EZH2 inhibitor at high concentrations, at low concentrations it proliferates faster than untreated P8T organoids, resulting in a high AUC value, but a relatively low IC50 value (measured with respect to the original viability). P20T on the other hand shows a typical resistant profile, with little response at all concentrations and no enhanced proliferation at low concentrations, which resulted in a relatively lower AUC value, but high IC50 (Supplementary Figure 2A). Interestingly, enhanced growth at low GSK126 concentrations was observed for more organoid lines than P8T. This suggests that these organoids benefit from mild reduction of cellular EZH2 activity. These observations are not entirely surprising, as the optimal level of EZH2 activity is context dependent. For instance, as opposed to its more classical role as an oncogene, *EZH2* appears to repress development of myelodysplastic syndrome [12, 34, 35]. In addition, both complete inactivation of PRC2 and overexpression of *Ezh2* stimulate progression of KRas-dependent lung tumors and do so in different ways [16]. Importantly, this stresses the importance of optimal dosing for EZH2 inhibition in cancer. Likewise, studies that correlated expression of PcG members with CRC patient prognosis have yielded contrasting results [20, 36–38], which indicates that also in CRC, PcG proteins may exert tumor suppressive as well as oncogenic actions depending on the mutational and transcriptional context. The dynamics in response to EZH2 inhibition that we observed will help us in further elucidating the conditions that define these actions in CRC.

We also analyzed prolonged inhibition of EZH2 in eight CRC organoid lines. To our surprise, there were more organoids lines that became more sensitive than those that became more resistant after prolonged treatment. This may be due to the accumulating dysregulation of the transcriptional program, as more and more H3K27me3 repressive marks are removed – and not replaced – from the chromatin over time, although the reduced viability might also have been caused by the dissociation to single cells at the start of passage 2. Alternatively, cells with decreased EZH2 activity may have decreased tumor-initiating potential, resulting in a reduction of outgrowing organoids in passage 2. Interestingly, cellular depletion of the PcG protein BMI1 was demonstrated to reduce stem cell-properties of CRC cells [39], which may suggest that PcG stabilizes stemness in some CRCs. Further, we did not see in any of the eight organoid lines in passage 2 a sudden growth acceleration indicative of a transition to a state at which cells benefit from low EZH2 levels. However, as this transition might require more time to develop or interaction with the microenvironment, adverse effects on patient survival because of prolonged EZH2 inhibition [15] can still be an issue in CRC.

Our further exploration of potential associations between the published organoid properties and GSK126 response yielded a number of associating features. We have found that *ATRX*-mutant organoid lines are sensitive to GSK126 treatment. *ATRX* is frequently mutated in tumor cells with alternative lengthening of telomeres (ALT), and its loss has been shown to promote ALT [40–42]. It is possible that cancer cells with ALT rely on PRC2 function, and that therefore inactivation of both ATRX and EZH2 is synthetic lethal in CRC. As EZH2 has previously been shown to be involved in DNA damage repair [43, 44], it may help cells to cope with replicative stress at ALT telomeres.

Mutation of *PAX2* was also found to associate with sensitivity to EZH2 inhibition. The capacity of PAX2 to stimulate gene expression is lost when associated with GRG4 during lineage specification and leads to PRC2 recruitment to PAX2 target genes [45]. *PAX2* mutation may therefore cause a shift in PRC2 target genes, which would promote tumor progression. Sudden PRC2 inactivation by GSK126 treatment could then cause derepression of target genes, which is detrimental to cell survival or leads to cell differentiation.

Further, our analysis yielded an inverse correlation between GSK126 response and *BIK* expression. It is conceivable that organoids with high *BIK* levels are more susceptible to apoptotic signals - for instance through p53 signaling due to EZH2 inhibition.

Of all the drug response associations studied, response to EZH2 inhibition correlated most strongly with response to Nutlin-3a. Nutlin-3a specifically affects p53-WT cells and in this panel of human CRC organoids, p53 mutation was previously found to associate with resistance to Nutlin-3a [18]. The observed responses to GSK126 in our study do not correlate with p53 mutation status, however, which is likely due to other mechanisms that inactivate p53 signaling. We therefore examined the response to EZH2 ablation in a genetically less disrupted background and demonstrated that the expression of WT p53 renders cells responsive to EZH2 inactivation. This response is likely triggered in combination with an oncogenic impulse, because normal intestinal tissue and small intestinal organoids are not affected by EZH2 ablation (Koppens et al. [46] and data not shown). This may suggest that EZH2 suppresses oncogene-induced senescence in intestinal tissue by keeping p53 signaling in check, which could be achieved by Polycomb-mediated repression of *CDKN2A* or of target genes of p53 itself [47].

Here, we demonstrate how organoids derived from different colon tumors are affected by EZH2 inhibition and highlight several molecular features that associate with response. It will be of interest to investigate if other types of cancer depend on the same genetic factors in their response to EZH2 inhibition. The results of our study demonstrate the potential of EZH2 inhibitors as therapeutics in the treatment of CRC patients.

## MATERIALS AND METHODS

### GSK126 response assays using the 3D organoid culture system

Organoids were gently digested to single cells using TrypLE Express (Life technologies) and counted. Cells were resuspended in Matrigel (Corning) and seeded onto 48-well plates with a concentration of 2000 cells in 25 μL Matrigel per well. 250 μL of complete medium was then added, which consisted of basal medium (Advanced DMEM/F12 supplemented with Pen/Strep (Life technologies), Glutamax and 10 mM HEPES) supplemented with 1x B27 supplement (Life technologies), 1.25 mM N-acetyl-L-cysteine (Sigma-Aldrich), 50 ng/mL EGF (Life technologies), 100 ng/mL Noggin (Peprotech), 10% Rspo1-conditioned medium, 10 mM Nicotinamide (Sigma-Aldrich), 500 nM A-83-01 (Tocris) and 10 μM SB202190 (Sigma-Aldrich). Three days after seeding, when single cells had formed small organoids, the medium was refreshed for medium with different GSK126 concentrations. Medium was refreshed every three days. When further growth of organoids at any concentration became compromised – this time differed between organoid lines (Supplementary Table 1F) – the organoids were digested to single cells with TrypLE and cells were counted using a Bürker counting chamber. We regarded conditions as being growth-compromising when either: the organoids had grown to such a close vicinity that further growth was hampered, or the culture medium increasingly turned acidic, or organoids started to produce large amounts of detached cells. For eight CRC organoid lines, single cells of each condition were again seeded at 2000 cells per well and the same GSK126 treatment as before passaging was continued. Selection of these passage 2 organoid lines was solely based on having a relatively medium-to-fast growth rate. All viability experiments were performed in triplicate or more.

### GSK126 treatment of adherently cultured human CRC organoids

Culture plates were coated with 15 μg/mL of Poly-ornithine (Sigma-Aldrich) overnight at 37°C, and then washed three times with water. The plates were subsequently coated with laminin (Sigma-Aldrich) for at least three hours. Organoids were gently digested to single cells using TrypLE Express, counted and seeded on laminin-coated culture plates directly after laminin was removed. Medium was refreshed every two to three days. Cells were passaged by trypsinizing with TrypLE Express, counting, spinning down and reseeding on laminin-coated culture plates. The medium used was complete medium supplemented with 10 μM Y27632 (Merck). Upon initiation of GSK126 treatment, 0.25 * 10^6^ cells were seeded per well in a 12-well plate and treated with either DMSO or 4 μM of GSK126 for two weeks. Cells were counted at the end and during each passaging step to calculate the relative expansion.

### *Ezh2* knockdown in murine tumor organoids

293T cells were transfected with lentiviral packaging plasmids and pFH1t vectors containing sequences encoding either a short hairpin with random sequence or a short hairpin against *Ezh2*. Per construct, 4 10-cm plates were transfected and refreshed one day after. Two and three days after transfection, taps were taken, which were then ultracentrifuged at 20,000 rpm for 2 h. Combined pellets of both taps were resuspended in Advanced DMEM/F12 supplemented with Pen/Strep (Life technologies), Glutamax and 10 mM HEPES) supplemented with 1x B27 supplement (Life technologies), 1× N2 supplement (Life Technologies), 1.25 mM N-acetyl-L-cysteine (Sigma-Aldrich), 50 ng/mL EGF (Life technologies), 100 ng/mL Noggin (Peprotech), Polybrene (Millipore) and Y27632. The organoids that were subsequently infected with this virus were originally derived from small intestines of *VillinCre;Apc*^LoxP/LoxP^*;Kras*^G12D/+^ (VAR) and *VillinCre;Apc*^LoxP/LoxP^*;Kras*^G12D/+^*;Trp53*^R172H/-^ (VAPR) mouse models by the lab of Owen Sansom. VAR organoids were also further compounded by retroviral transduction of a pRetrosuper-sh-p53 construct (VAR-*sh-Trp53*). Organoids were trypsinized using TrypLE (Life technologies) to single cells, combined with virus and centrifuged at 600rpm and 32°C for 1 h. The cells were then incubated for 6 h at 37°C after which they were seeded in Matrigel (Corning) and overlaid with complete medium supplemented with Y27632. Two days after infection, medium was refreshed and Puromycin was added. Infected organoids were kept on this selection antibiotic for two weeks. The organoids were then treated with Doxycycline to induce expression of short hairpins.

### Western blotting

Western blotting was performed as described by Koppens et al. [46]. The following primary antibodies were used: anti-EZH2 (BD Biosciences), anti-H3K27me3 (Abcam, AB64850), anti-p53 (Monosan, Monx10194) and anti-Vinculin (Sigma-Aldrich, V9131).

### Calculating AUCs and IC50s

Viability was calculated in two different ways: using a piecewise linear approximation as well as continuous curve fitting. This resulted in four different measures of response: two types of area under the curve (AUC) and two corresponding types of IC50 (concentration at 50% viability). The piecewise linear AUC is simply the trapezoidal area under the dose response curve. The corresponding IC50 is interpolated from the segment which contains the 50% viability. In all cases, the AUC and IC50 were computed for each replicate independently, and then the median was taken over all replicate AUCs and replicate IC50s respectively. These are shown in Figure 1D–1G.

To model the peaks at the beginning of the eight-point response curve, for some samples, we combined a two-parameter normal density function with a two-parameter sigmoid function and estimated a total of five parameters using nonlinear least squares. These parameters include mean and standard deviation for the normal density, shift and slope for the dose response and a proportionality coefficient which scales the normal density component contribution in the fit. All replicates (three to four per organoid line) were taken into account in the estimation. For three samples (P7T, P28T in passage 1, P14T in passage 2) the model did not converge because for these fast responders even the two-parameter sigmoid curve is not a good model: the two-parameter sigmoid is not very steep at zero concentration for most parametrizations. For the fit of these samples we took the minimum sum of squares of the residuals solution over 500 parameter samplings. The fitted curves were used to obtain fitted AUCs and fitted IC50s. These are shown in Supplementary Figure 2A and 2B.

### Processing molecular data

For this analysis, the gene expression data (for all 22 samples) and mutation data (for all 22 samples) from the original publication [18] were used, and matched to 20 samples of the 22 (two samples were not distributed by the original biobank). The gene expression data was downloaded as normalized originally (using rma-sketch, within Affymetrix Power Tools [18]), and row/gene mean-centered.

The mutation data provided by Supplementary Table 1J from ref. [18] was filtered to exclude mutations in intergenic regions, introns, silent mutations, mutations in non-coding regions, UTRs and flank regions. In addition, 12 genes, frequently mutated in and associated with hereditary CRC (*APC*, *BMPR1A*, *MLH1*, *MSH2*, *MSH6*, *MUTYH*, *PMS2*, *POLD1*, *POLE*, *PTEN*, *SMAD4* and *STK11*), were screened for presence of pathogenic germline single nucleotide variants, using the raw exome sequencing data [18]. This resulted in the inclusion of a *MUTYH* mutation (rs587782885) for P25T and *APC* mutations for P18T (rs62619935) and P20T (rs137854573) to the list of somatic mutations in Supplementary Table 1J from ref. [18]. Mutations were then summarized as binary values per gene, zero for wild type and one for mutated. In this analysis, no distinction was made between different types of mutation in the same gene.

To analyze differences between consensus molecular subtypes (CMS) in terms of *EZH2* expression, we compared the expression in 239 out of 461 RNA-seq samples from the TCGA database (Level 3 RNA-seq data, frozen tissue, labelled COAD (Colon adenocarcinoma), from both the Illumina GA and Illumina HiSeq platforms). These 239 samples are such that both CMS classifiers from [17] assign a valid subtype and agree on the classification. *EZH2* expression comparison between the different subtypes was done via a *t*-test.

### Associating molecular features with response

For associating CRC molecular subtypes with response, we made use of the subtyping (developed by Sadanandam et al. [21]) of the panel of organoids as presented in the original study [18].

For both gene expression data and the mutation data two types of association analyses were performed. The first was univariate: associating single features with response, followed by multiple test correction. For the mutation data we employed *t*-tests of the viability of wild type samples versus the viability of mutant samples (adjusted *p*-value < 0.25). For gene expression, we looked at the correlation of expression of single genes with drug response (|cor| > = 0.5).

For the second arm of analysis, we looked for associations using all features, and due to the small sample size, also using a smaller selected number of features. For gene expression the subset was the top 11% of high-varying genes (2430 genes with standard deviation above 0.7). For mutations, a list of 93 selected mutations, specific for colon cancer was used, as derived in Iorio et al, [22].

### Comparing monotonous to non-monotonous responders

To study the phenomenon of non-monotonicity in response in some of the organoids, samples were split into monotonic and non-monotonic (the latter being P17T, P19TB, and P8T in passage 1 and P18T and P31T in passage 2, see Supplementary Figure 2A and 2B) based on the criterion that there should be a viability increase of at least 50% for the fitted curve at the lowest nonzero concentration. In terms of mutations the two groups were tested for differences using a chi-square test for each mutation. To detect differences in gene expression, we used the standard procedure within the limma R package.

## SUPPLEMENTARY MATERIALS




