# Supplementary material for: Large variety in a panel of human colon cancer organoids in response to EZH2 inhibition
Source: Oncotarget. 2016 Sep 13;7(43):69816–28. doi: 10.18632/oncotarget.12002 (PMC5342517; doi:10.18632/oncotarget.12002)
Supplement: Supplementary file 1 [file oncotarget-07-69816-s001.pdf]

Large variety in a panel of human colon cancer organoids in response to EZH2 inhibition

Supplementary Materials

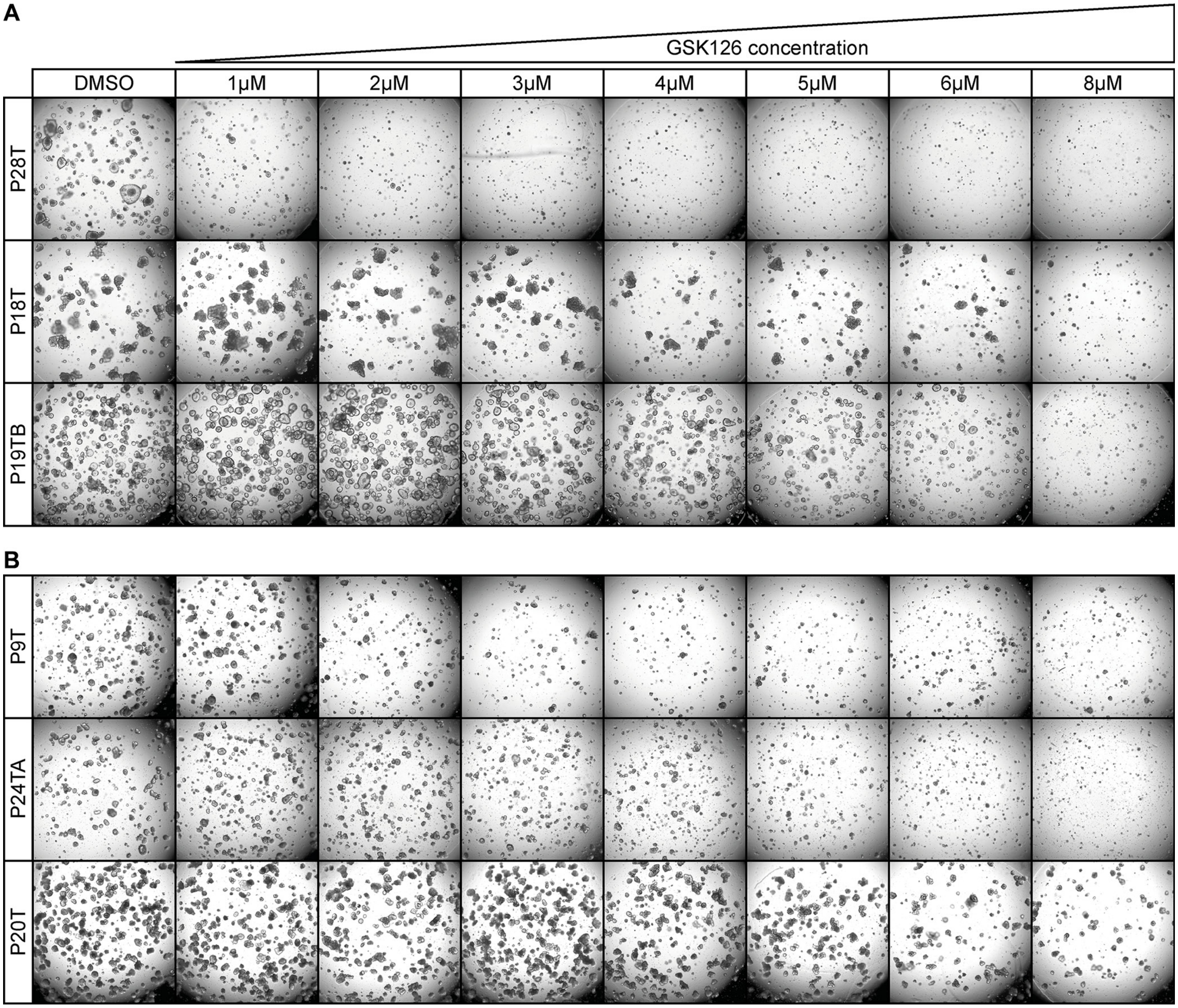

Supplementary Figure S1: Different growth responses of CRC organoid lines to GSK126 treatment. (**A–B**) Representative images of examples of very sensitive, moderately resistant and very resistant organoid lines in passage 1 (A) and passage 2 (B).

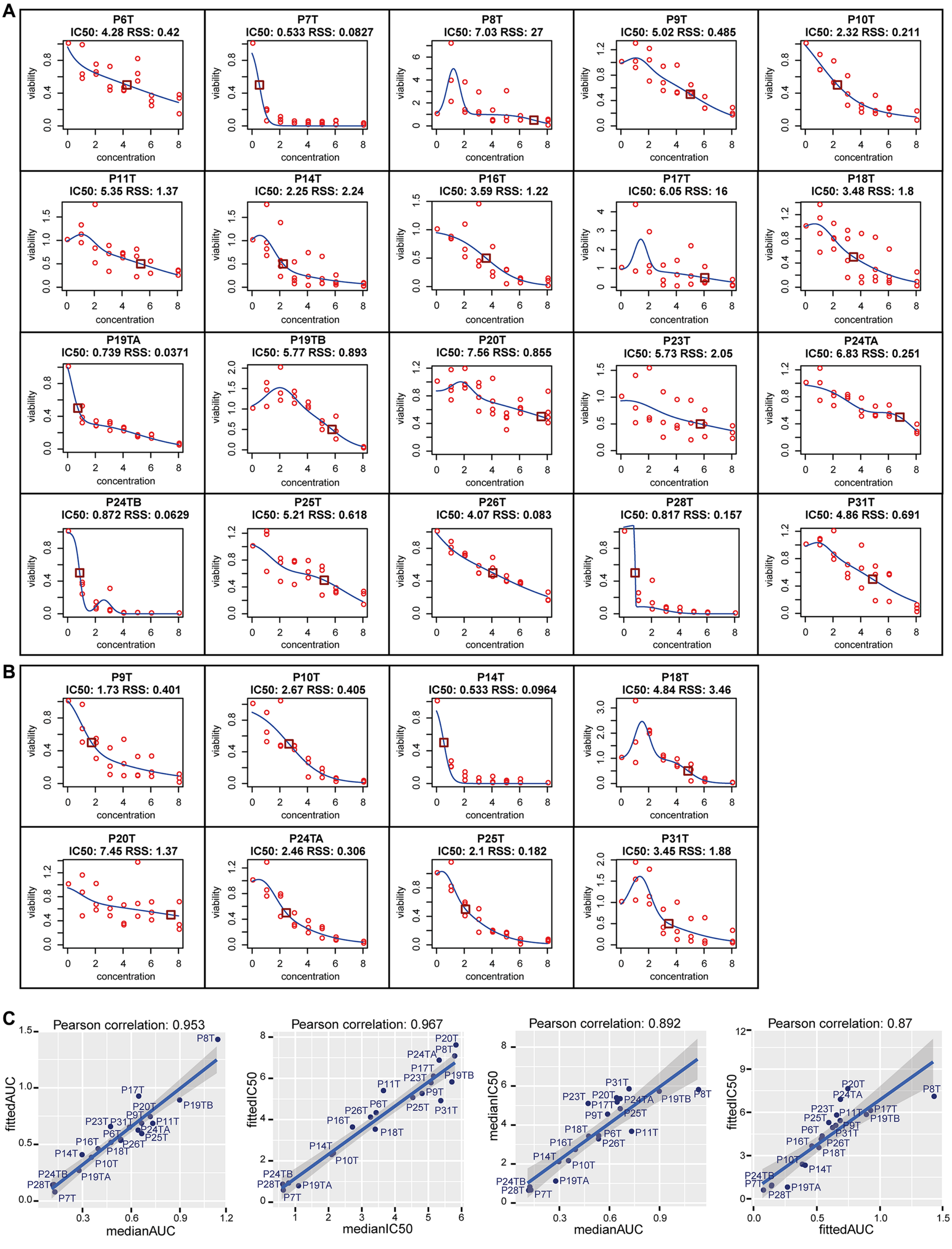

Supplementary Figure S2: Computation of GSK126 response through different methods for all tested organoids. (**A**) Passage 1 viability plots of all replicates for each organoid line. Curves are fitted and IC50 is demarcated on the curve with a red square. (**B**) Passage 2 viability plots with fitted curves of all replicates for each organoid line. (**C**) Comparison of the different methods to compute AUC and IC50.

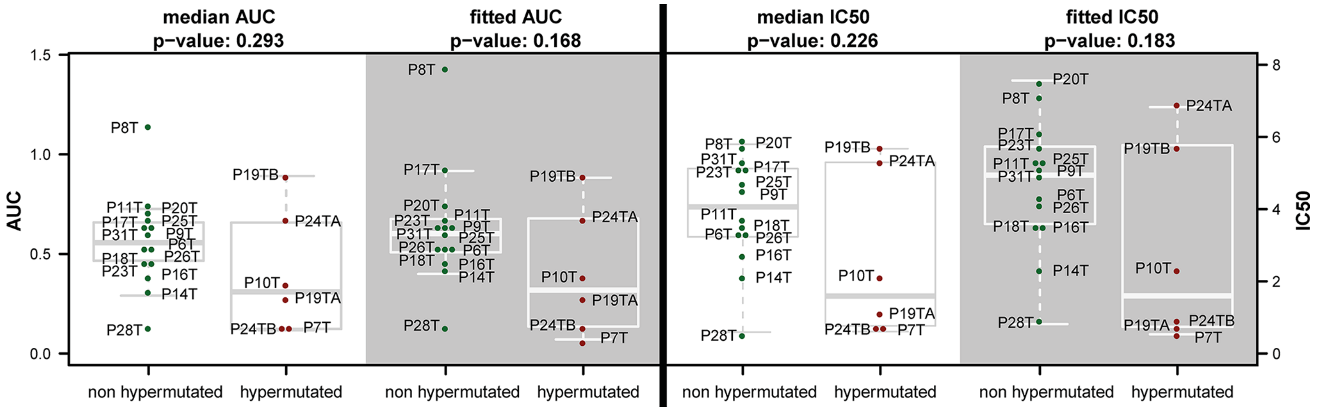

Supplementary Figure S3: Weak association between hypermutation status and response to EZH2 inhibition. Hypermutation status does not significantly associate with GSK126 response using any of the viability measures because two out of six hypermutants are resistant to the drug. Four box plots (AUC: left two panels, IC50: right two panels; median: white background, fitted: grey background) comparing non-hypermutated organoids (left group, green dots) with hypermutated organoids (right group, red dots). Horizontal lines within boxes demarcate the median; boxes delineate the middle 50% of the data; and whiskers mark 25% and 75% quartiles.

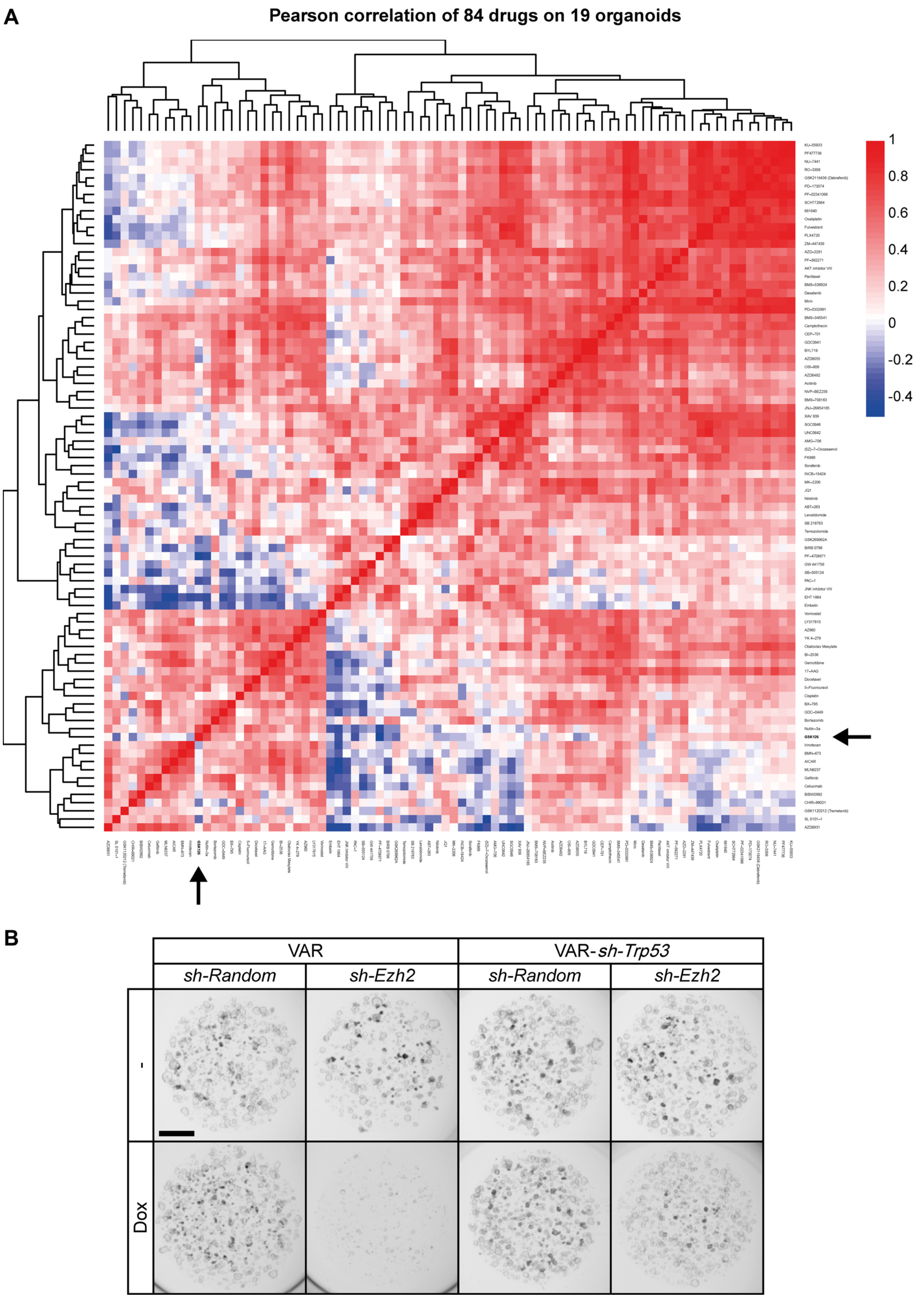

Supplementary Figure S4: Correlations of 83 previously tested drugs and GSK126. (**A**) Heatmap of Pearson correlations between all pairs of 84 drugs (83 from the Sanger panel [18] and GSK126), hierarchically clustered based on correlation distance. The color of each cell in the heatmap reflects the Pearson correlation between its two corresponding drugs, according to the color scale on the right (blue - negative, white - zero, red - positive). Arrows point to GSK126. (**B**) Macroscopical images of VAR and VAR-*sh-Trp53* organoids with and without *Ezh2* knockdown. Organoid growth is rescued in *Ezh2*-knockdown organoids by *Trp53* knockdown. Scale bar: 2 mm, same magnification applies to all images in B.

Supplementary Table S1: (**A**) **List of genes that are differentially expressed between monotonous and non-monotonous responding organoids.** Viabilities from passage 1 were used. (**B**) Summary of subtyping according to four classifiers for all organoid samples. (**C**) List of genes whose mutation status associated with degree of response to GSK126 in passage 1. This list results from analysis of all mutations present in the panel of organoid lines. (**D**) List of genes whose mutation status associated with degree of response to GSK126 in passage 1. This list results from analysis of 93 selected mutations associated with colon cancer [22]. (**E**) List of genes whose expression correlated with response to GSK126 treatment in passage 1. (**F**) Treatment times for the 20 organoid lines in passage 1 and for the 8 organoid lines in passage 2. See Supplementary_Table_S1
